# Untargeted metabolomics to evaluate antifungal mechanism: a study of *Cophinforma mamane* and *Candida albicans* interaction

**DOI:** 10.1007/s13659-022-00365-w

**Published:** 2023-01-03

**Authors:** Asih Triastuti, Marieke Vansteelandt, Fatima Barakat, Carlos Amasifuen, Patricia Jargeat, Mohamed Haddad

**Affiliations:** 1 UMR 152 Pharma Dev, IRD, UPS, Université de Toulouse, 31400 Toulouse, France; 2 Department of Pharmacy, Universitas Islam Indonesia, Yogyakarta, 55584 Indonesia; 3 Dirección de Recursos Genéticos y Biotecnología, Instituto Nacional de Innovación Agraria, Avenida La Molina 1981, La Molina, Lima, 15024 Peru; 4 Laboratoire Evolution et Diversité Biologique UMR 5174, CNRS, IRD, UPS, Université de Toulouse, 31062 Toulouse, France

**Keywords:** Metabolomics, Fungal co-culture, Anti-fungal, Virulence

## Abstract

Microbial interactions between filamentous fungi and yeast are still not fully understood. To evaluate a potential antifungal activity of a filamentous fungus while highlighting metabolomic changes, co-cultures between an endophytic strain of *Cophinforma mamane* (CM) and *Candida albicans* (CA) were performed. The liquid cultures were incubated under static conditions and metabolite alterations during the course were investigated by ultra-performance liquid chromatography–tandem mass spectrophotometry (UPLC–MS/MS). Results were analyzed using MS-DIAL, MS-FINDER, METLIN, Xcalibur, SciFinder, and MetaboAnalyst metabolomics platforms. The metabolites associated with catabolic processes, including the metabolism of branched-chain amino acids, carnitine, and phospholipids were upregulated both in the mono and co-cultures, indicating fungal adaptability to environmental stress. Several metabolites, including C20 sphinganine 1-phosphate, myo-inositol, farnesol, gamma-undecalactone, folinic acid, palmitoleic acid, and MG (12:/0:0/0:0) were not produced by CA during co-culture with CM, demonstrating the antifungal mechanism of CM. Our results highlight the crucial roles of metabolomics studies to provide essential information regarding the antifungal mechanism of *C. mamane* against *C. albicans*, especially when the lost/undetected metabolites are involved in fungal survival and pathogenicity.

**Graphical Abstract:**

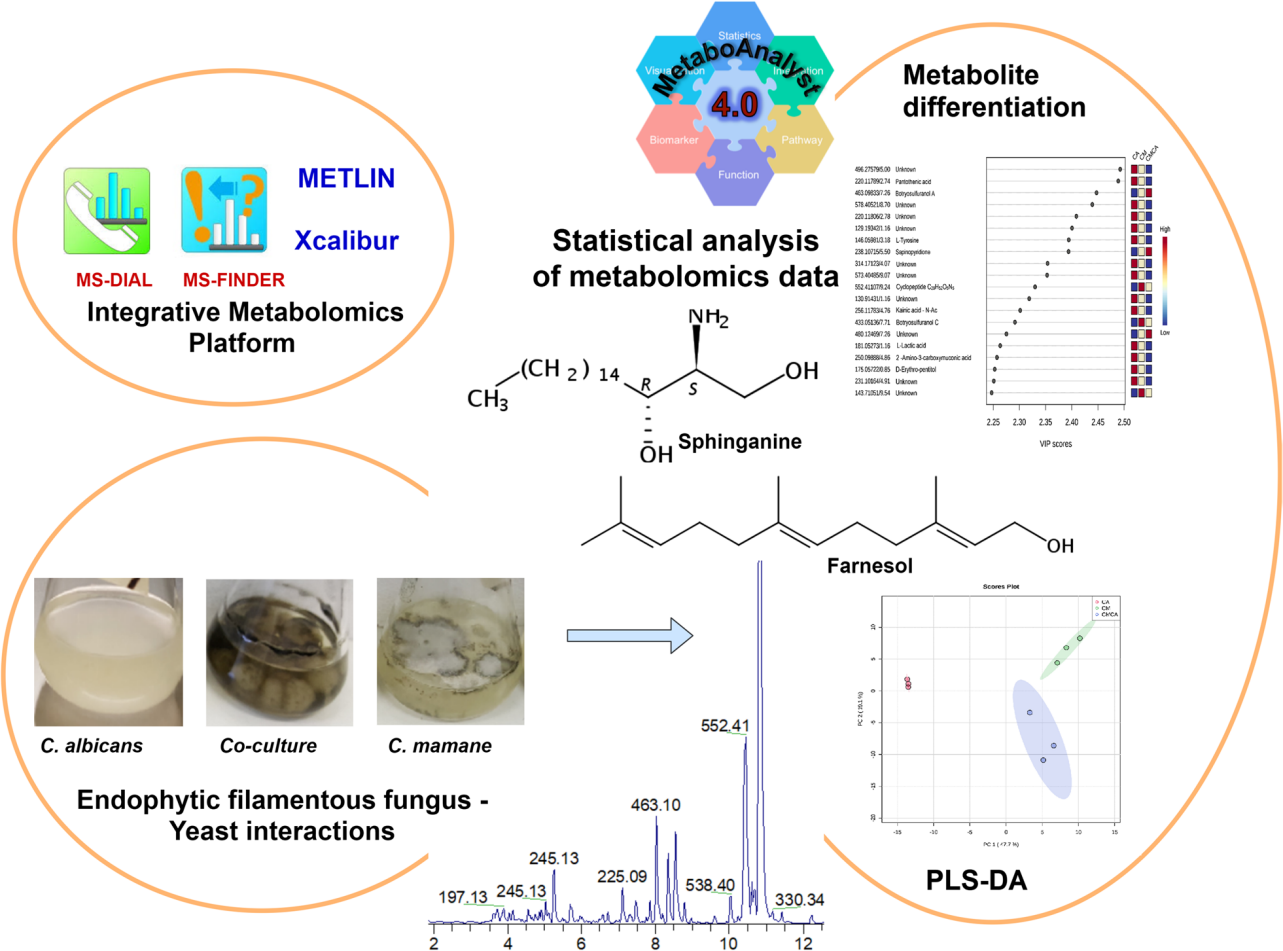

## Introduction

*Candida albicans* (CA) is an opportunistic yeast species present on the mucosal surfaces of the human gastrointestinal (GI), respiratory, and genitourinary systems that is frequently employed as a model organism for the investigation of human fungal pathogens. As a member of the human microflora, *C. albicans* is typically a harmless commensal fungus; however, it can exist as an opportunistic pathogen in immunocompromised or immunodeficient individuals [1]. The ability of *C. albicans* to infect such a variety of host niches is supported by a wide range of fitness attributes and virulence factors, including the morphological transition between the yeast and hyphal forms, the expression of adhesins and invasin proteins on the cell surface or thigmotropism [2], the capacity to respond and sense changes in the surface contours and the formation of biofilms [3], phenotypic switching, and the secretion of hydrolytic enzymes [2, 4, 5]. Moreover, *C. albicans* morphogenesis can be regulated by quorum sensing, a microbial communication mechanism. The primary quorum-sensing molecules include farnesol, tyrosol, farnesoic acid, and dodecanol [3, 6, 7].

*Cophinforma mamane* (CM) is a filamentous fungus known to produce thiodiketopiperazine alkaloids (botryosulfuranols A–C) and mellein derivates (*trans*-4-hydroxymellein, *cis*-4-hydroxymellein, and 5-hydroxymellein). Botryosulfuranols, previously isolated by our team, have been reported to be cytotoxic against several cancer cell lines [8] whereas mellein derivates have been reported to be active against *S. aureus* and methicillin-resistant *S. aures* [9] as well as specific fungi [10, 11]. In addition, the ethyl acetate extracts of *C. mamane* grown in potato dextrose broth (PDB) exhibited antifungal activity against *C. albicans* in our preliminary screening*.* These findings prompted us to investigate the mechanism by which *C. mamane* inhibits *C. albicans.* An agar-based antifungal test is commonly used as a preliminary antifungal screening method for natural products to evaluate antifungal activity by comparing the zone of inhibition of the candidates to that of the standard antifungal drug (ex. fluconazole) [12]. Although this method is simple and quick, the results cannot be used to infer the antifungal mechanism of the candidates. Study of the fungal metabolome approach employs ultra-performance liquid chromatography–tandem mass spectrophotometry (UPLC–MS/MS) to provide data on up and down-regulated metabolites. Thus, metabolites associated with fungal virulence/pathogenicity can be highlighted if detected by this method.

The goal of this study was to analyze the metabolite changes that occurred during the competitive interaction between CA and CM, using untargeted metabolomic approach, in order to potentially detect antifungal or quorum-sensing molecules. *C. albicans* was co-cultured with a strain of *C. mamane* isolated from *Bixa orellana*, in potato dextrose broth medium (PDB). Metabolomic analyses and annotations were performed using MSDIAL, MSFINDER, METLIN, Xcalibur, and SciFinder platforms. We intended to carry out all of the experiments in a liquid culture medium because it is homogeneous and allows for the most interactions between co-cultivated microorganisms when compared to solid culture, making it more reliable and reproducible [13]. The limitations of the fungal database are overcome by combining several analysis platforms such as MSDIAL, MSFINDER, METLIN, Xcalibur, and SciFinder to produce valid data. All of these experimental conditions should allow us to highlight the alterations of secondary metabolite biosynthesis in dual cultures under static conditions, as well as potentially detecting some antifungal or quorum-sensing compounds.

## Result and discussion

### Fungal co-culture morphologies

**Fig. 1:**
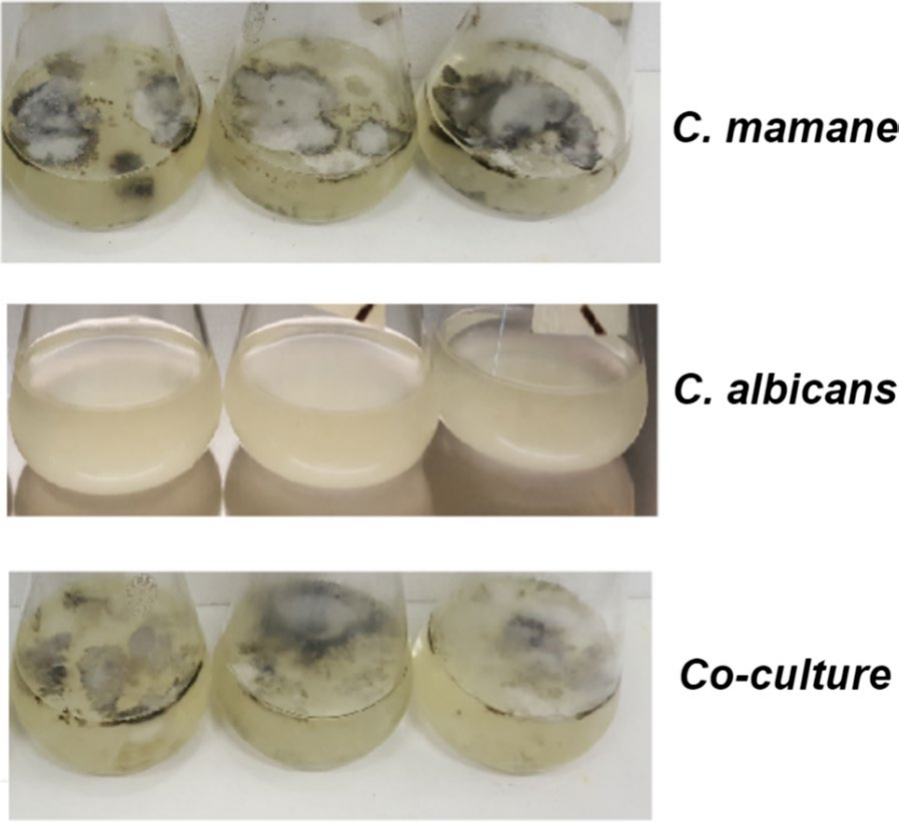
Morphological differences among cultures in PDB medium

*Cophinforma mamane* (CM), in presence of *C. albicans* (CA) or not, grew at the surface of the liquid medium and formed floating colonies (“mycelia mats”) with the top portion exposed to the air. CA, on the other hand, grew and adhered to the bottom of the flask while the medium remained clear, demonstrating typical biofilm production. The co-cultivation effect on fungal morphology appeared to mimic the mixed features of the two axenic cultures. Under static conditions, we observed floating colonies of CM with less pigmentation on top of the media and a biofilm of CA attached to the bottom of the Erlenmeyer flask, indicating no physical contact between the fungi (Fig. 1).

### Effects of co-culture in the fungal metabolomes

**Fig. 2:**
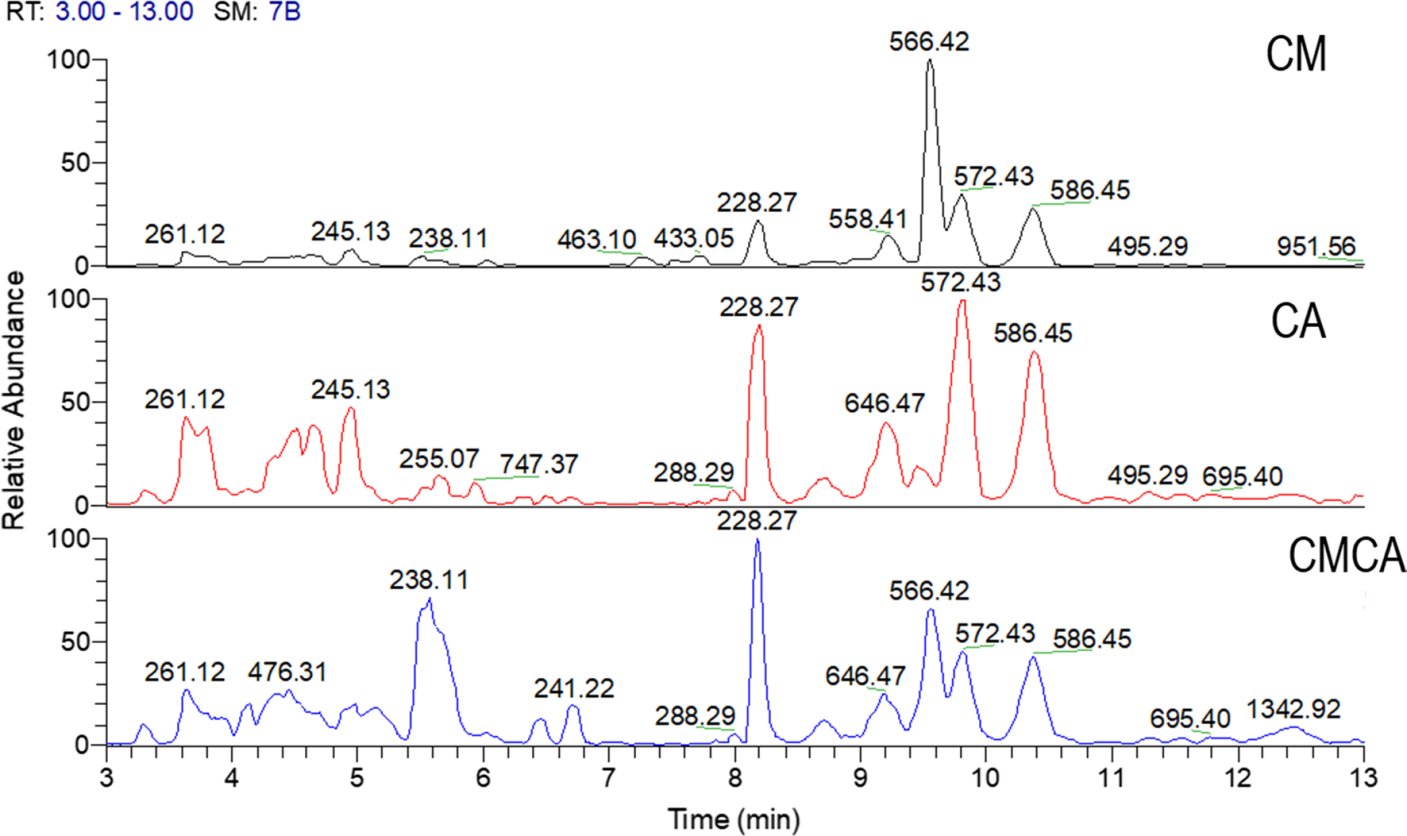
The UHPLC–ESI–HRMS chromatograms (positive ionization) of fungal cultures CM: *Cophinforma mamane*; CA: *Candida albicans*, CMCA: co-culture of CM and CA

Many chemical and physical factors contribute to the evolution of morphological forms of fungi. These can either directly or indirectly influence fungal metabolite production during submerged fermentation [14, 15]. Nevertheless, as metabolism changes could occur without any phenotypical modification, UHPLC–HRMS was used to compare the chemical profiles of fungi cultured in axenic vs co-culturing conditions [16, 17]. The resulting peak list from MS-DIAL was analyzed using MS-FINDER for the identification of putative fungal metabolites. A cross-confirmation using Xcalibur, METLIN, and SciFinder was then performed. A differential analysis to identify the up and downregulated metabolites in each culture was performed using MetaboAnalyst. The UPLC–HRMS base peak chromatograms from the axenic fungal cultures and their co-cultures are shown in Fig. 2.

**Fig. 3:**
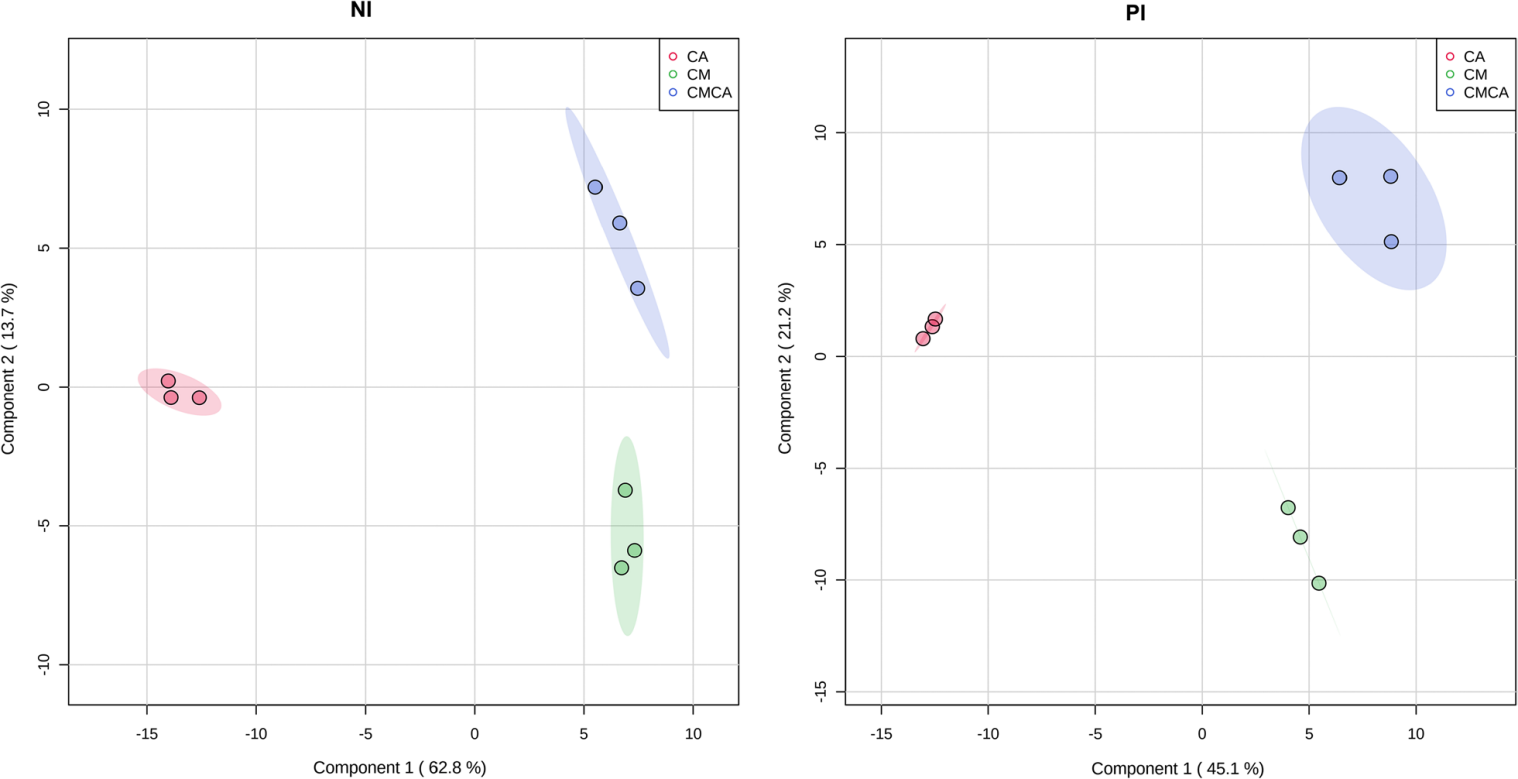
PLS-DA score plots of fungal cultures based on ESI− (NI) and ESI+ (PI) data obtained by UHPLC–HRMS (regional display of 95% confidence levels). CM: *Cophinforma mamane*; CA: *Candida albicans*, CMCA: co-culture of CM and CA

In multivariate group analysis, partial least squares (PLS)-discriminant analysis (DA) was used to differentiate groups and identify the intrinsic variations in the data sets. PLS-DA can analyze highly collinear and noisy data in a calibration model and provide a variety of useful statistics, such as accuracy of prediction and scores and loadings plots [18]. In this study, not surprisingly, PLS-DA revealed the presence of three different groups: CM, CA, and CMCA (Fig. 3). The culture condition determined the fungal metabolome in mono and co-cultures, as indicated by a clear separation among the groups.

**Fig. 4:**
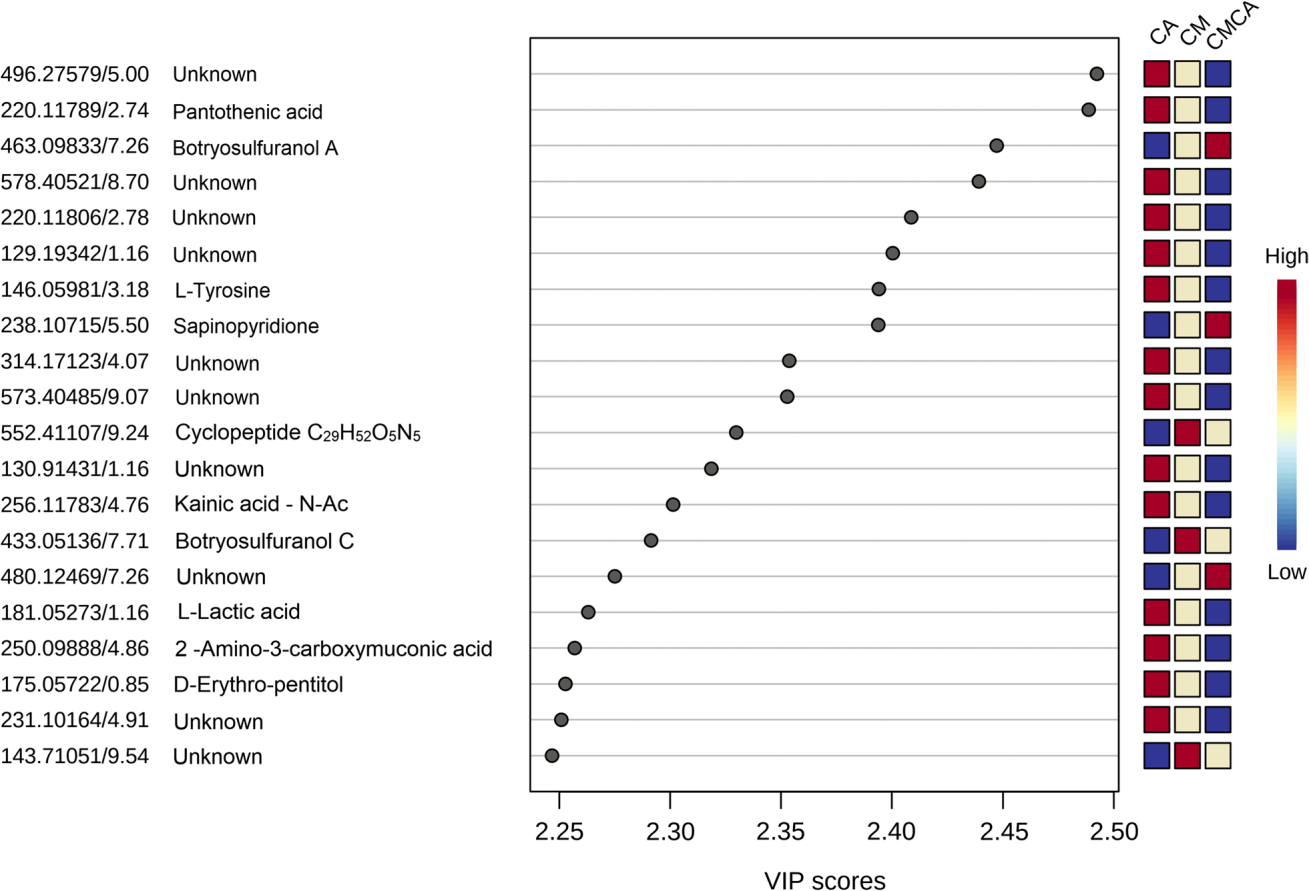
The top 20 compounds according to the VIP score (p < 0.05) responsible for metabolite differentiation among cultures. The boxes on the right indicate the relative concentrations of each metabolite in each group. CM: *Cophinforma mamane*; CA: *Candida albicans*, CMCA: co-culture of CM and CA

Using variable importance in projection (VIP) score and analysis of variance (ANOVA, p < 0.05), the most significant characteristics responsible for metabolite differentiation during interaction were identified. Figure 4 displays the top 20 metabolites verified by VIP score that were validated using the MSFINDER fungal database, SciFinder, and METLIN.

The two thiodiketopiperazines botryosulfuranols A and C, and a compound annotated as a cyclopeptide C_29_H_52_O_5_N_5_ produced by *C. mamane* were included among the 20 components. Interestingly, it seems that several compounds produced by *C. albicans* in monocultures are detected with a much lower intensity in co-culture with CM. It seems that several unknown compounds (undetected with HRMS database) produced by CA are no longer produced during co-culture. On the contrary, two compounds produced by CM (botryosulfuranol A and sapinopyridione) in monoculture are highly detected in co-culture, suggesting a potential role of these compounds in the antifungal mechanism of CM. Botryosulfuranol A is a member of thidikeopiperazines [8], and is an important fungi-derived chemical with antifungal action [19, 20]. Sapinopyridione was isolated from *Sphaeropsis sapinea* and demonstrated antimycotic activity [21].

### Prediction of the antifungal mechanism of *C. mamane*

Evaluation of the metabolites produced by axenic *C. albicans* and that were not detected in co-culture with *C. mamane* (Table 1) provides support for the hypothesis that *C. mamane* employs an inhibitory mechanism. The presence or absence of these compounds initially identified in the data matrix was manually confirmed using Xcalibur.

**Table 1:** Compounds in *C. albicans* that were not detected in the co-culture with *C. mamane* and their putative identification

*Observed m/z*/RT (min)	Molecular formula	Δ mass error (ppm)	Putative identification
137.1321/8.3	C_10_ H_16_	2.1876	7-Methyl-3-methylene-1,6-octadiene
203.1794/6.45	C_15_H_22_	0.1329	Gamma-undecalactone
220.1178/2.74	C_9_H_17_NO_5_	0.4543	Panthotenic acid
223.2056/7.98	C_15_H_26_O	0.1881	Farnesol
255.1954/8.85	C_15_H_26_O_3_	0.2782	Palmitoleic acid
259.2267/6.72	C_15_H_30_O_3_	0.2738	Tetradecanedioic acid
275.2215/5.91	C_15_H_30_O_4_	0.6758	MG (12:0/0:0/0:0)
354.155/4.40	C_17_H_23_NO_7_	0.7652	Dihydro zeatin riboside
410.3048/11.73	C_27_H_39_NO_2_	0.1364	C20 sphinganine 1-phosphate
474.1902/4.37	C_23_H_27_N_3_O_8_	6.7294	Folinic acid
538.1848/4.98	C_32_H_27_NO_7_	2.283	6-*O*-(2-amino-2-deoxy-alpha-d-glucosyl)-1d-Myo-inositol 1-(6-mercaptohexyl)phosphate
718.5804/12.75	C_40_H_80_NO_7_P	8.1870	PC (P-18:0/14:0)

+ Detected in culture/− not detected in culture; RT, retention time

Several metabolites that are essential for the survival and virulence of *C. albicans* including C20 sphinganine 1-phosphate, myo-inositol, farnesol, gamma-undecalactone, and MG (12:/0:0/0:0) were not produced during co-culturing with *C. mamane*. Sphinganine is a simple sphingolipid precursor involved in the synthesis of complex sphingolipids [22]. The loss of C20 sphinganine 1-phosphate may affect the activities of the sphingolipid biosynthetic pathway, resulting in sensitivity to *C. mamane*. As previously reported [23], the upregulation of the sphingolipid phytosphingosine 1-phosphate enhanced the efflux of miconazole drugs to reduce the sensitivity of *C. albicans*. In our study, the downregulation of sphinganine possibly increased its sensitivity to the antifungal activity of *C. mamane* in co-culturing.

Myo-inositol, also known as inositol, is an essential nutrient that is used in the synthesis of phosphatidylinositol. Phosphatidylinositol not only acts as a structural component of the membrane, but it also functions as a precursor for several other essential lipid molecules, such as sphingolipids, ceramides, and glycosylphosphatidyl [24, 25]. Myo-inositol is essential for the growth and virulence of *C. albicans*, which can meet this need by de novo biosynthesis [26]. A reduction or loss of myo-inositol in co-culturing may indicate that the mechanism of *C. mamane* disrupts the *C. albicans* membrane.

In addition, when the two strains were co-cultured, the quorum-sensing molecule farnesol, which is produced by *C. albicans*, was depleted. Farnesol affects genes involved in drug resistance, cell wall maintenance, phagocytic response, surface hydrophobicity, and iron transport [27]. Farnesol inhibits Ras-1 adenylate cyclase protein A kinase signaling pathway which leads to repression of the hyphal growth, stress response, metabolism, and drug resistance. A recent study stated farnesol also affects the ABC efflux transporters, changing *C. albicans* resistance to azole [7]. The absence of farnesol impairs *C. albicans*’ ability to maintain cell surface hydrophilicity and respond to cell-stress mechanisms caused by the presence of *C. mamane* in the same medium [28, 29]. Furthermore, gamma-undecalactone and MG (12:0/0:0/0:0) were also not detected in the coculture of *C. albicans* and *C. mamane*. Gamma-decalactone and MG (12:0/0:0/0:0) exist in the cell membrane and are responsible for the metabolism of fatty acids in the membrane.

PLS-DA results showed that, in co-culturing, most of these metabolites were produced by *C. mamane*, thereby corroborating the hypothesis that *C. mamane* inhibited metabolites production by CA during fungal interaction. Thus, *C. mamane* may suppress *C. albicans* metabolite production in co-culturing by directly synthesizing compounds that destroy or perturb the growth of *C. albicans* or through a competitive mechanism that limits the access of *C. albicans* to nutrients. This finding highlights the usefulness of metabolome analysis to elucidate the antifungal mechanisms of *C. mamane* against *C. albicans*.

To the best of our knowledge, studies on co-culturing yeast and filamentous fungi, especially those dedicated to the fungal metabolome exploration, are extremely limited. Moreover, there has been no publication on the co-culturing of *C. albicans* and filamentous fungi in submerged cultures. For example, a study by Pereira et al. [30] proposed the co-culturing of fungi and yeast in a solid medium to detect antimicrobial activities, and García-Martínez et al*.* [31] studied co-cultures of the yeasts *Saccharomyces cerevisiae* and *Penicillium chrysogenum*, but none of these studies applied metabolomics to further understand the mechanisms of interactions.

## Experimental section

### Fungal strains

*Cophinforma mamane* E224 strain was isolated from *Bixa orellana* leaves and identified using barcoding (PCR amplification of the ITS with ITS5 and ITS4 primers) [32], sequencing, and sequence comparison with GenBank databases). *C. mamane* was was taken in Iquitos (national reserve of Allpahuayo Mishana, Amazonian rainforest) in November 2013, GPS coordinates: 3° 58′ 02.3 S, 73° 25′ 03.9 W). In the UMR 152 Pharmadev laboratory collection, the strain was cryopreserved in 30% glycerol at − 80 °C. This strain was procured in compliance with all applicable laws and regulations. The *C. mamane* sequence can be found in the GenBank database (accession number MG457709). *C. albicans* ATCC 90028 strain was obtained from Thermo Fisher Scientific France (Illkirch-Graffenstaden, France) and was propagated in Sabouraud’s agar media according to the supplier’s protocol.

### Fungal culture and extraction

In this study, *C. mamane* (CM), *C. albicans* (CA), and co-culture of *C. mamane* and *C. albicans* (CMCA) were grown in 100-mL Erlenmeyer flasks containing 50 mL of PDB for 7 days at 27 °C. Ultrasonication at 20 kHz for 1 h in 50 mL of ethyl acetate was used to extract the entire PDB culture. The mycelia in the culture supernatant were removed using Miracloth^®^ (EMD Millipore Corporation, Billerica, MA, USA). After liquid–liquid extraction, the organic phases were dried on anhydrous MgSO_4_, filtered, and concentrated under reduced pressure. A flask containing a PDB medium without any fungal culture was used as the control medium.

### UHPLC–HRMS profiling

All crude extracts (Ultimate 3000; Thermo Fisher Scientific) were profiled on the UHPLC–DAD–LTQ Orbitrap XL instrument according to a modified version of a previous study [33]. Dried crude extracts were dissolved in methanol until a final concentration of 2 mg/mL. The samples (2 µL) were loaded onto a C18 column with a guard column (100 2.1 mm, internal diameter, 1.7 m, Waters, MA, USA). An equal mixture of each replicate was prepared in the quality control (QC) group. The QC-all group, which consisted of an equal mixture of all samples, was also prepared to evaluate the system’s stability over the time required for the analysis of the entire sample set.

The Waters system with a flow rate of 0.3 mL/min was used for the UHPLC analysis. With a gradient elution (0–0.5 min, 95% A; 0.5–12 min, 95% −5% A; 12–15 min, 5% A; 15–15.5 min, 5% −95% A; 15.5–19 min, 95% A), the mobile phase consisted of solvent A (0.1% formic acid–water) and solvent B (0.1% formic acid-acetonitrile). Positive and negative ionization modes were used to detect mass with an electrospray source set to 15,000 resolving power (full width at half maximum at 400 m/z). The mass range scanned was 100–1500 Da. The ISpray voltage was set to 4.2 kV (positive mode) and 3.0 kV (negative mode) and the capillary temperature was 300 °C (negative mode). Before the experiment began, the mass measurement was calibrated externally. After each full MS scan, data-dependent MS/MS was performed using stepped collision-induced dissociation (CID) on the three most intense peaks.

### Peak analysis

MS-DIAL (data-independent MS/MS deconvolution for comprehensive metabolome analysis) version 4.90 was utilized to process the UHPLC–HRMS data [34]. Automatic feature detection was performed between 0.3 and 13 min, with positive and negative mode mass signal extraction ranging from 100 to 1500 Da. MS1 and MS2 tolerances were set to 0.01 and 0.025 Da, respectively, in centroid mode. The optimal detection threshold for MS1 was set at 2 105, and for MS2 it was set at 10. The peaks list from MS-DIAL was then transferred to Excel to generate a matrix of m/z ratios, retention times, and peak intensities. Using the MetaboAnalyst 5.0 metabolomics data analysis and interpretation software [35], the matrix was transformed into a comma-separated value format. If the sample peak appeared in at least two of the three sample peaks, it was retained. Adducts were analyzed and eliminated from the peak list. The matrix was cleared of all uncultured media peaks.

### Putative compound identification

MS-FINDER version 3.52 (PRIMe: Platform for RIKEN Metabolomics; http://prime.psc.riken.jp/compms/msfinder.html) was used to predict the production of metabolites [36] restricted to fungal compounds (CRC Press v26:2). The results are presented as a list of compounds ranked by the score value of the match. This value represented the uncertainty in the precise mass, the isotopic pattern score, and the experimental MS/MS fragmentation based on the in-silico matches. Only structures with a score greater than seven were retained for further inspection. SciFinder and METLIN were used to cross-reference the identification of putative fungal compounds.

## Conclusions

The present study on the co-culture of *C. mamane* and *C. albicans* may be the first such study on filamentous fungi-yeast interaction using untargeted metabolomics. Metabolomics analysis is crucial for learning about antifungal mechanisms when screening antifungal agents, particularly when the detected metabolites are linked to fungal survival and pathogenicity. In this work, *C. mamane* inhibited the synthesis of compounds that are crucial for the survival and virulence of *Candida albicans* such as C20 sphinganine 1-phosphate, myo-inositol, farnesol, gamma-undecalactone, and MG (12:/0:0/0:0)*.* This inhibition could be related to the up-regulated production of some compounds produced by *C. mamane* such as botryosulfuranol A which could exert an inhibitory effect on some biosynthetic pathway of *C. albicans*. Thus, this research highlights the utility of metabolome analysis in understanding the antifungal processes of a putative fungus or chemical against pathogenic microbes. Isolating metabolites produced by *C. mamane* to evaluate their action against *C. albicans* and to proceed further in elucidating this inhibitory activity will be the focus of future experiments.

## References

[1] Dadar M, Tiwari R, Karthik K, Chakraborty S, Shahali Y, Dhama K (2018). *Candida albicans*—biology, molecular characterization, pathogenicity, and advances in diagnosis and control—an update. Microb Pathog.

[2] Mayer FL, Wilson D, Hube B (2013). *Candida albicans* pathogenicity mechanisms. Virulence.

[3] Cavalheiro M, Teixeira MC (2018). Candida biofilms: threats, challenges, and promising strategies. Front Med.

[4] Desai J (2018). *Candida albicans* hyphae: from growth initiation to invasion. J Fungi.

[5] De Sordi L, Mühlschlegel FA (2009). Quorum sensing and fungal-bacterial interactions in *Candida albicans*: a communicative network regulating microbial coexistence and virulence. FEMS Yeast Res.

[6] Verbeke F, De Craemer S, Debunne N, Janssens Y, Wynendaele E, Van de Wiele C, De Spiegeleer B (2017). Peptides as quorum sensing molecules: measurement techniques and obtained levels *in vitro* and *in vivo*. Front Neurosci.

[7] Černáková L, Dižová S, Gášková D, Jančíková I, Bujdáková H (2019). Impact of farnesol as a modulator of efflux pumps in a fluconazole-resistant strain of *Candida albicans*. Microb Drug Res.

[8] Barakat F, Vansteelandt M, Triastuti A, Jargeat P, Jacquemin D, Graton J, Mejia K, Cabanillas B, Vendier L, Stigliani J-L (2019). Thiodiketopiperazines with two spirocyclic centers extracted from *Botryosphaeria mamane*, an endophytic fungus isolated from *Bixa orellana* L. Phytochemistry.

[9] Pongcharoen W, Rukachaisirikul V, Phongpaichit S, Sakayaroj J (2007). A new dihydrobenzofuran derivative from the endophytic fungus *Botryosphaeria mamane* PSU-M76. Chem Pharm Bull.

[10] Hussain H, Jabeen F, Krohn K, Al-Harrasi A, Ahmad M, Mabood F, Shah A, Badshah A, Rehman NU, Green IR (2015). Antimicrobial activity of two mellein derivatives isolated from an endophytic fungus. Med Chem Res.

[11] Mitaka Y, Mori N, Matsuura K (2019). A termite fungistatic compound, mellein, inhibits entomopathogenic fungi but not egg-mimicking termite ball fungi. Appl Entomol Zool.

[12] Berkow EL, Lockhart SR, Ostrosky-Zeichner L (2020). Antifungal susceptibility testing: current approaches. Clin Microbiol Rev.

[13] Elisashvili V (2012). Submerged cultivation of medicinal mushrooms: bioprocesses and products. Int J Med Mushrooms.

[14] Cui YQ, Van Der Lans RGJM, Luyben KCAM (1997). Effect of agitation intensities on fungal morphology of submerged fermentation. Biotechnol Bioeng.

[15] Papagianni M (2004). Fungal morphology and metabolite production in submerged mycelial processes. Biotechnol Adv.

[16] Guijas C, Montenegro-Burke JR, Warth B, Spilker ME, Siuzdak G (2018). Metabolomics activity screening for identifying metabolites that modulate phenotype. Nat Biotechnol.

[17] Lee WNP (2006). Characterizing phenotype with tracer based metabolomics. Metabolomics.

[18] Gromski PS, Muhamadali H, Ellis DI, Xu Y, Correa E, Turner ML, Goodacre R (2015). A tutorial review: metabolomics and partial least squares-discriminant analysis—a marriage of convenience or a shotgun wedding. Anal Chim Acta.

[19] Kajula M, Ward JM, Turpeinen A, Tejesvi MV, Hokkanen J, Tolonen A, Häkkänen H, Picart P, Ihalainen J, Sahl HG (2016). Bridged epipolythiodiketopiperazines from *Penicillium raciborskii*, an endophytic fungus of *Rhododendron tomentosum*. J Nat Prod.

[20] Iwasa E, Hamashima Y, Sodeoka M (2011). Epipolythiodiketopiperazine Alkaloids: total syntheses and biological activities. Isr J Chem.

[21] Evidente A, Fiore M, Bruno G, Sparapano L, Motta A (2006). Chemical and biological characterisation of sapinopyridione, a phytotoxic 3,3,6-trisubstituted-2,4-pyridione produced by *sphaeropsis sapinea*, a toxigenic pathogen of native and exotic conifers, and its derivatives. Phytochemistry.

[22] Cowart LA, Obeid LM (2007). Yeast sphingolipids: recent developments in understanding biosynthesis, regulation, and function. Biochim Biophys Acta.

[23] Vandenbosch D, Bink A, Govaert G, Cammue BPA, Nelis HJ, Thevissen K, Coenye T (2012). Phytosphingosine-1-phosphate is a signaling molecule involved in miconazole resistance in sessile *Candida albicans* Cells. Antimicrob Agents Chemother.

[24] Jin JH, Seyfang A (2003). High-affinity myo-inositol transport in *Candida albicans*: substrate specificity and pharmacology. Microbiology.

[25] Michell RH (2008). Inositol derivatives: evolution and functions. Nat Rev Mol Cell Biol.

[26] Reynolds TB (2009). Strategies for acquiring the phospholipid metabolite inositol in pathogenic bacteria, fungi and protozoa: making it and taking it. Microbiology.

[27] Polke M, Leonhardt I, Kurzai O, Jacobsen ID (2018). Farnesol signalling in candida albicans—more than just communication. Crit Rev Microbiol.

[28] Alem MAS, Oteef MDY, Flowers TH, Douglas LJ (2006). Production of tyrosol by candida albicans biofilms and its role in quorum sensing and biofilm development. Eukaryot Cell.

[29] Han TL, Cannon RD, Villas-Bôas SG (2011). The metabolic basis of *Candida albicans* morphogenesis and quorum sensing. Fungal Genet Biol.

[30] Pereira E, Santos A, Reis F, Tavares RM, Baptista P, Lino-Neto T, Almeida-Aguiar C (2013). A new effective assay to detect antimicrobial activity of filamentous fungi. Microbiol Res.

[31] García-Martínez T, Peinado RA, Moreno J, García-García I, Mauricio JC (2011). Co-culture of *Penicillium chrysogenum* and *Saccharomyces cerevisiae* leading to the immobilization of yeast. J Chem Technol Biotechnol.

[32] White TJ, Bruns T, Lee S, Taylor J. Amplification and direct sequencing of fungal ribosomal rna genes for phylogenetics. In: PCR protocols: a guide to methods and applications; 1990; pp. 315–322.

[33] Triastuti A, Vansteelandt M, Barakat F, Trinel M, Jargeat P, Fabre N, Amasifuen Guerra CA, Mejia K, Valentin A, Haddad M (2019). How histone deacetylase inhibitors alter the secondary metabolites of *Botryosphaeria mamane*, an endophytic fungus isolated from *Bixa orellana*. Chem Biodivers.

[34] Tsugawa H, Cajka T, Kind T, Ma Y, Higgins B, Ikeda K, Kanazawa M, VanderGheynst J, Fiehn O, Arita M (2015). MS-DIAL: data-independent ms/ms deconvolution for comprehensive metabolome analysis. Nat Methods.

[35] Pang Z, Zhou G, Ewald J, Chang L, Hacariz O, Basu N, Xia J (2022). Using metaboanalyst 5.0 for LC–HRMS spectra processing, multi-omics integration and covariate adjustment of global metabolomics Data. Nat Protoc.

[36] Tsugawa H, Kind T, Nakabayashi R, Yukihira D, Tanaka W, Cajka T, Saito K, Fiehn O, Arita M (2016). Hydrogen rearrangement rules: computational ms/ms fragmentation and structure elucidation using MS-FINDER software. Anal Chem.

